# Preparing Thick Gradient Surface Layer in Cu-Zn Alloy via Ultrasonic Severe Surface Rolling for Strength-Ductility Balance

**DOI:** 10.3390/ma15217687

**Published:** 2022-11-01

**Authors:** Qisheng Sun, Jiapeng Sun, Yantao Fu, Bingqian Xu, Ying Han, Jianqing Chen, Jing Han, Hao Wu, Guosong Wu

**Affiliations:** 1 College of Mechanics and Materials, Hohai University, Nanjing 211100, China; 2 Key Laboratory of Advanced Structural Materials, Changchun University of Technology, Changchun 130012, China; 3 School of Mechanical and Electrical Engineering, China University of Mining and Technology, Xuzhou 221116, China; 4 Guangdong Key Laboratory of Materials and Equipment in Harsh Marine Environment, Guangzhou Maritime University, Guangzhou 510725, China

**Keywords:** Cu-Zn alloy, gradient structure, microstructure, ductility

## Abstract

A gradient structure (GS) design is a prominent strategy for strength-ductility balance in metallic materials, including Cu alloys. However, producing a thick GS surface layer without surface damage is still a challenging task limited by the available processing technology. In this work, a gradient structure (GS) surface layer with a thickness at the millimeter scale is produced in the Cu-38 wt.% Zn alloy using ultrasonic severe surface rolling technology at room temperature. The GS surface layer is as thick as 1.1 mm and involves the gradient distribution of grain size and dislocation density. The grain size is refined to 153.5 nm in the topmost surface layer and gradually increases with increasing depth. Tensile tests indicate that the single-sided USSR processed alloy exhibits balanced strength (467.5 MPa in yield strength) and ductility (10.7% in uniform elongation). Tailoring the volume fraction of the GS surface layer can tune the combination of strength and ductility in a certain range. The high strength of GS surface layer mainly stems from the high density of grain boundaries, dislocations and dislocation structures, deformation twins, and GS-induced synergistic strengthening effect. Our study elucidates the effect of the thick GS surface layer on strength and ductility, and provides a novel pathway for optimizing the strength-ductility combination of Cu alloys.

## 1. Introduction

Cu-Zn alloys, i.e., brass, are one type of the oldest structure metallic materials due to an excellent combination of mechanical properties, corrosion resistance, wear resistance, and machinability [1]. Now, brasses are still in widespread popularity in many industrial applications for producing essential parts and components, such as gears, spring, valve element, etc. Therefore, continuously improving the strength and ductility of the brasses is valuable in engineering and thus a consistent pursuit for scientists and engineers.

It is well known that the strength-ductility trade-off is a long-standing dilemma in the field of structural materials [2,3]. This dilemma is also outstanding for Cu and its alloys, and thus extraordinary efforts have been devoted to strengthening Cu alloys with less sacrificing ductility [4,5,6,7]. Architecting gradient structure (GS) is a success story to overcome this dilemma for various alloys [8,9,10,11,12,13,14] and has been successfully applied in pure Cu and several Cu alloys [15,16,17,18,19]. Lu et al. prepared a gradient nano-grained pure Cu using a surface mechanical grinding treatment (SMGT) [10]. In this gradient nano-grained Cu, the nano-grained surface layer was confined by the coarse-grained substrate, and thus strain localization, which was the main reason for the very limited tensile ductility of nano-grained metals, was effectively suppressed under tension. Therefore, the gradient nano-grained pure Cu is twice as strong as the coarse-grained pure Cu, while its ductility was almost not degraded. SMGT can also be used on the Cu-Al alloys to achieve the gradient nanostructure and improves the strength [20]. Zhang et al. introduced a gradient structured layer in the H62 brass through a surface spinning strengthening technology [21]. The gradient brass showed a slightly improved tensile yield strength, but a decreased ultimate tensile strength. Lu et al. applied laser shock peening (LSP) on the H62 brass to enhance the tensile properties [22]. LSP not only induced the nanograins with a very small size of 10 nm in the topmost surface, but also led to surface amorphization at localized areas, and thus simultaneously improved the strength and ductility of the brass. In these studies, the generated GS surface layer in Cu alloys is generally less than 500 μm in thickness and its volume fraction is very small. The thin and low volume-fraction GS surface layer enhances the yield strength of the whole sample without serious scarifying ductility, while the ultimate tensile strength shows a slight improvement, and even decreases.

The superior strength-ductility balance within high strength scope is also expected in many industrial applications. Li et al. prepared a GS layer with a thickness of 1.45 mm in the Cu-30Zn plate (3.6 mm in thickness) using a combined processing route of friction stir processing and rotationally accelerated shot peening, which led to a substantial improvement in both yield strength and ultimate tensile strength [23]. Therefore, the GS Cu alloys with thicker GS surface layer is extremely attractive for achieving high strength. Unfortunately, it is still generally difficult to prepare a thick GS surface layer in the Cu alloys with high strength due to their poor work hardening capacity and few available processing technologies, although it is relatively easy to achieve in pure Cu [24,25,26] and annealed Cu alloys with low strength [27]. In GS metals, the GS surface layer is the main origin of the high strength due to their refined grains and high-density lattice defects [8,20]. The thick GS surface layer enhances the volume fraction and thus leads to higher strength. Moreover, Li et al. found that the thick GS layer facilitated a high synergetic strengthening effect and contributed to extra yield strength [23]. However, the deformation mechanism of the GS Cu alloy with a thick GS surface layer is still far from clear. More processing technologies for thick GS surface layers and related formation mechanisms are expected, and an in-depth understanding of the microstructure, mechanical behavior, and deformation mechanism of the Cu alloys with a thick GS surface layer is needed to further optimize the strength-ductility combination and realize their full potential in applications.

Ultrasonic severe surface rolling (USSR) is a newly developed technology to prepare a thick GS surface layer and has been applied to selective laser melted 316L stainless steel and ZE41 Mg alloy for superior strength and ductility [28,29]. The USSR technology exhibits advanced capabilities of effectively producing large-scale, geometrically complex samples with GS surface layers with a smooth surface. In this study, a thick GS surface layer was prepared in the Cu-38 wt.% Zn alloy plate by USSR at room temperature to achieve a good strength-ductility balance. The microstructure evolution of the GS surface layer in a depth range of 0–750 μm was systematically characterized by electron backscatter diffraction (EBSD) and transmission electron microscope (TEM). The mechanical behavior of the single-sided and double-sided USSR-processed Cu-Zn alloy was investigated compared to the as-received alloy. The underlying mechanism behind the superior mechanical properties induced by the thick GS surface layer was also discussed. This study provides a further understanding of the microstructure and mechanical behavior of the Cu alloys with a thick GS surface layer and offers a pathway for designing high-performance Cu alloys.

## 2. Materials and Methods

The commercial Cu-38 wt.% Zn alloy (H62 brass) was used and machined into the plate-like sample with a size of 50 × 50 × 2 mm followed by grinding. Then, the USSR process was conducted on the surface of the as-received sample for three passes, during which a rolling tungsten carbide tip (14 mm in diameter) attached to an ultrasonic device scanned the surface of the plate-shaped sample line by line with a static force. The main process parameters included a static load of 1200 N, scanning velocity of 0.02 m/s, feed interval of 0.1 mm, and frequency of 28 kHz. The single-sided USSR processed sample is hereafter named the SS-USSR sample, and the double-sided USSR processed sample is named the DS-USSR sample. The USSR induces severe plastic deformation and thus reduces the thickness of the processed sample.

The microstructure of the as-received sample was characterized by EBSD (Oxford Instrument C-Nano). The microstructure of the USSR samples was observed by longitudinal section EBSD and TEM (FEI, Talos, F200X). For TEM observation of the USSR sample, a 500-μm-thick slice was cut along the longitudinal section, mechanically ground, and then ion milled around the processed surface (PIPS II, Gatan 695). The SS-USSR specimens for EBSD were prepared through grinding, mechanical polishing, and ion polishing. The Channel 5 software (version, 5.0.9.0) was used to analyze the EBSD data. High angular grain boundaries with misorientation angle *θ* over 15° and (111) twins were identified, and the kernel average misorientation (KAM) was calculated, which reflected the density of the lattice defect mainly composed of dislocations. The mechanical properties were measured by tensile tests at room temperature with a rate of 0.5 mm/min on an electronic universal testing machine (SUNS UTM4204X). The dog-bone-shaped sample with an initial gauge length of approximately 7.5 mm and width of approximately 2 mm was machined by a wire-cut electrical discharge machining and then ground on the machining surface. Tensile tests of each type of sample were independently repeated two times to ensure data reproducibility. Tensile specimens of the free-standing topmost 350-μm-thick gradient layer were prepared from the whole SS-USSR tensile sample by cutting away other layers using a wire-cut electrical discharge machining and grinding. The micro-hardness meter (HYS-1000Z) was used to measure the micro-hardness variation along the depth of the USSR sample at a load of 0.98 N and a duration time of 15 s. The hardness at each depth is the average value of three measurements.

## 3. Results

### 3.1. Microstructure

**Figure 1:**
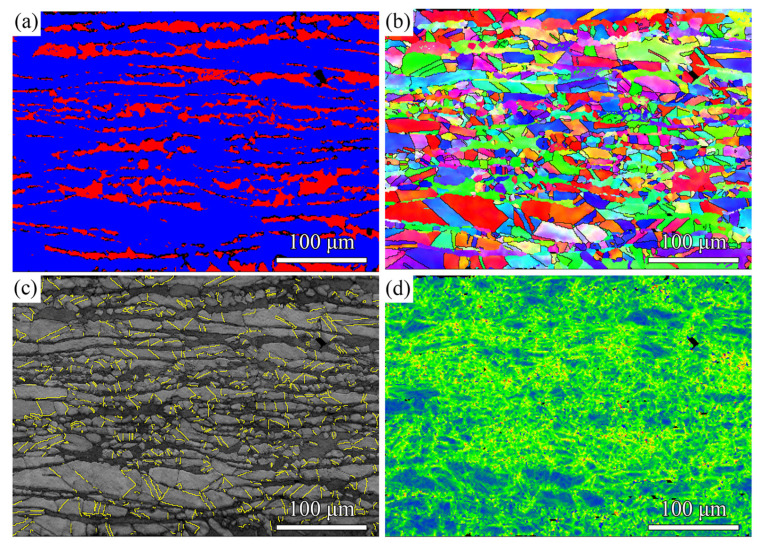
EBSD result of the as-received sample: (**a**) phase map; (**b**) IPF map; (**c**) band contrast map with twin boundaries; (**d**) KAM map. The yellow lines in the Panel c highlight the twin boundaries.

Figure 1 shows the EBSD results of the as-received sample. The Cu-Zn alloy is a typical dual-phase alloy composed of *α*-Cu_0.64_Zn_0.36_ phase and *β*-CuZn phase, which is confirmed by the EBSD phase map shown in Figure 1a. The volume fractions of *β* phase are 19.3%. The as-received alloy has a laminar microstructure with arranged alternately *α* phase and *β* phase, as indicated by the EBSD inverse pole figure (IPF) map and phase map (Figure 1b). The *α-*phase laminae have a thickness of 12.0 μm, and the *β-*phase laminae are 6.2 μm in thickness. The average grain size of the as-received sample is 12.5 μm measured by the line intercept method. Some twins are observed within *α*-phase grains, as shown in Figure 1c. The KAM map indicates that the as-received sample has a high KAM value, manifesting high defect density.

**Figure 2:**
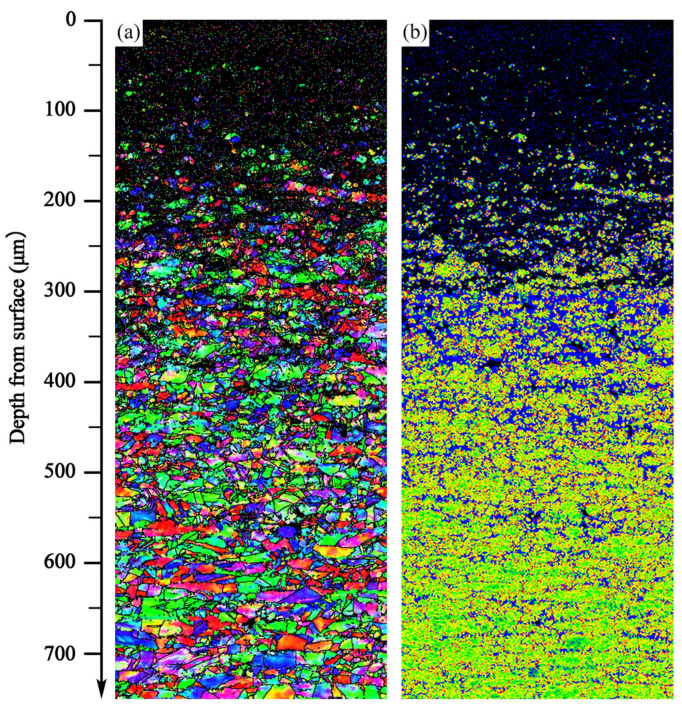
Typical longitudinal section EBSD result of the SS-USSR sample from the top surface: (**a**) IPF map, (**b**) KAM map.

**Figure 3:**
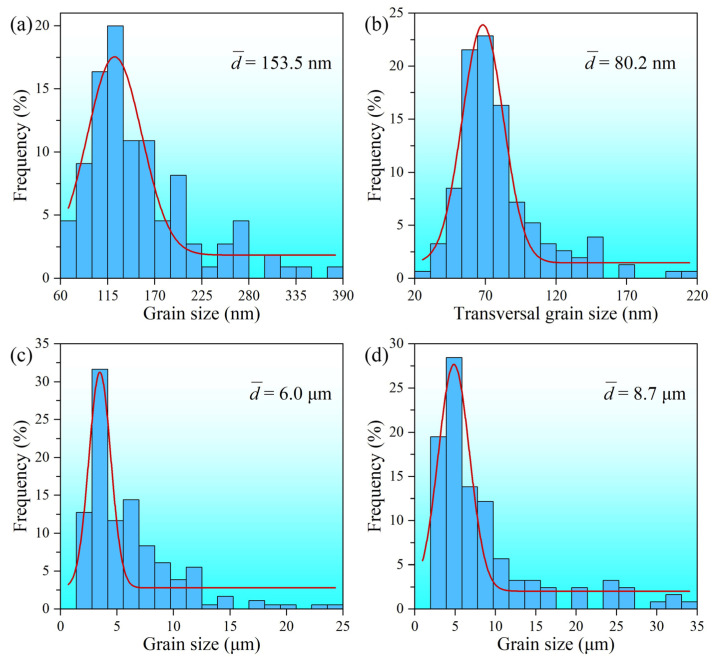
Distribution of structure size at the depth of (**a**) 0 μm; (**b**) 50 μm; (**c**) 300 μm; and (**d**) 700 μm.

Figure 2 presents the longitudinal section EBSD result of the 750-μm-thick surface layer of the SS-USSR sample. In the depth range of 200 to 750 μm, the microstructure is featured by refined grains embedded with high-density dislocations. The grains are irregular and their size gradually increases from 6.0 μm to 8.7 μm with increasing depth from 300 μm to 700 μm, as shown in Figure 3c,d. Some twins are still visible, but no obvious gradient change is perceived. Very high KAM value indicates high dislocation density in this layer, as depicted in Figure 2b. The microstructure in the topmost 200-μm-thick surface layer is significantly refined and hardly identified through EBSD. This result indicates that the thickness of the GS surface layer in the USSR alloy is over 750 μm. Therefore, the DS-USSR sample has a sample-level GS. Our previous studies indicated that USSR generates a high gradient strain and strain rate, and thus induces a thicker GS [28].

**Figure 4:**
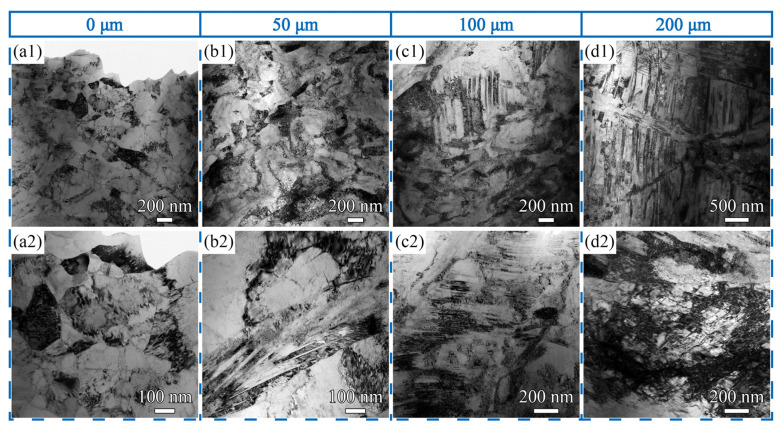
Typical longitudinal section TEM images of the SS-USSR sample at depth of (**a1**) 0 μm; (**b1**) 50 μm; (**c1**) 100 μm; and (**d1**) 200 μm. (**a2**–**d2**) are high-magnificent image corresponding to the depth of 0 μm, 50 μm, 100 μm, and 200 μm, respectively.

The microstructure evolution of the SS-USSR sample along depth in the topmost 200 μm-thick surface layer is further characterized through longitudinal section TEM observation, and the results are shown in Figure 4. In the topmost surface layer, nearly equiaxed ultrafine *α-*phase and *β-*phase grains are observed as well as some elongated ultrafine grains, as indicated by Figure 4(a1,a2) and Figure 3a. The average grain size is 153.5 nm. Twins and high-density dislocations are also visible in some grains. In the depth of 50 μm, the microstructure is mainly featured by the elongated ultrafine grains and dislocation structure as well as a few equiaxed ultrafine grains, as shown in Figure 4(b1,b2). The elongated ultrafine grains have an average transversal grain size of 80.2 nm (Figure 3b) and are decorated with dense dislocations and dislocation structures. Twin lamellae are also detected in some grains. Increasing depth to 100 μm, the elongated ultrafine grains and/or subgrains coexist with enlarged grains embedded with dense twin lamellae, as depicted in Figure 4(c1,c2). The twin lamellae are 41.6 nm in thickness and sustain a high density of dislocations. Most of the twin boundaries are curved but not straight. The curved twin boundaries are induced by the accumulation of dislocations at twin boundaries [30]. With the further increase of the depth to 200 μm, the feature of the microstructure changes to the twin lamellae with high-density dislocations, as Figure 4(d1,d2) show. The twin lamellae are 57.3 nm in thickness. High-density dislocations and dislocation structures are also observed, as indicated in Figure 4(d2). Present microstructure characterization suggests that dislocation subdivision and twin fragmentation are simultaneously activated for refining grains during the USSR processing of the Cu-38Zn alloy. The detailed operation of the two refinement mechanisms can be found in the references [31,32].

### 3.2. Mechanical Properties

**Figure 5:**
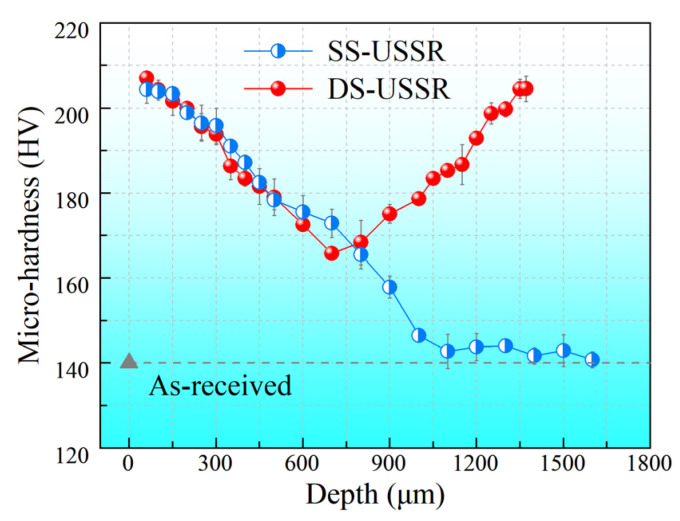
Distribution of micro-hardness along the increasing depth from the topmost surface. For the DS-USSR sample, the depth increases from the first USSR processed side.

Figure 5 presents the distributions of micro-hardness along the increasing depth from the top surface for the SS-USSR and DS-USSR samples. The as-received sample has a homogeneous mico-hardness of 140 HV through the longitudinal section. After the USSR processing, the microhardness at a depth of 60 μm is as high as 207.1 HV, which is 1.5 times higher than that of the as-received sample. The micro-hardness of the SS-USSR sample decreases monotonically with increasing depth and tends to a steady value at the depth of 1.1 mm, indicating that the USSR process can produce a thick GS surface layer. The DS-USSR sample shows a V-shaped hardness profile due to the double-sided USSR process. The micro-hardness of the DS-USSR sample in the core reaches 165.7 HV, which is 1.2 times as high as that of the as-received alloy. Thus, the DS-USSR sample is covered wholly by the GS surface layer.

**Figure 6:**
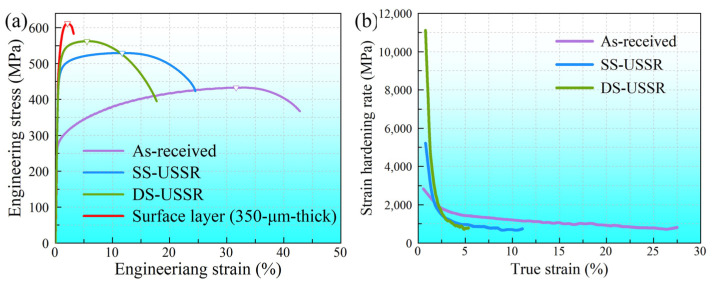
(**a**) Tensile stress–strain curves of the as-received, SS-USSR, DS-USSR samples as well as a 350-μm-thick surface layer; (**b**) Strain hardening rate as a function of true strain of the as-received, SS-USSR, DS-USSR samples.

Figure 6a presents the typical tensile stress–strain curves of the SS-USSR and DS-USSR samples as compared to the as-received sample, and the mechanical properties are collected in Table 1. It is evident that USSR greatly improves the strength of the Cu-38Zn alloy. The DS-USSR sample exhibits the highest yield strength of 476.2 MPa and ultimate tensile strength of 553.6 MPa, and still holds a reasonable ductility (16.9% in elongation and 5.2% in uniform elongation). The yield strength of the DS-USSR sample is 1.6 times as high as that of the as-received sample. The SS-USSR sample has a slightly decreased yield strength of 464.7 MPa and ultimate tensile strength of 530.3 MPa as well as greatly increased ductility (22.5% in elongation and 10.7% in uniform elongation) as compared to the DS-USSR sample. For comparison, the stress–strain curve of the 350-μm-thick surface layer is also measured. This thin surface layer is much strong but low-ductility, whose mechanical behavior is similar to the nano-grained metallic materials widely reported in the references [33,34]. Moreover, the DS-USSR and SS-USSR samples exhibit improved work-hardening capacity at a low true strain (less than 6%) compared to the as-received sample, as indicated in Figure 6b. In comparison, the DS-USSR sample shows a relatively high work hardening capacity as compared to the SS-USSR sample at a low true strain. Increasing strain, the work-hardening rate of the DS-USSR and SS-USSR samples rapidly decreases below that of the as-received sample, leading to reduced ductility.

## 4. Discussion

Many studies have shown that the Cu alloys with homogenous nanocrystalline (NC) and ultrafine-grained (UFG) structure exhibit excellent strength as compared to their coarse-grained counterparts, but disappointing ductility. For example, the uniform elongation of the UFG Cu-32Zn alloy with an average grain size of 91 nm is just 1.7% while its yield strength is over 700 MPa [35,36]. Low ductility greatly limits the wide application of the NC and UFG Cu alloy. Comparatively, the present SS-USSR and DS-USSR samples exhibit better strength-ductility balance.

Based on our microstructure observation and previous literature [37,38], the enhanced strength of the DS-USSR and SS-USSR samples mainly originates from the individual contribution of the structural components and GS-induced synergistic strengthening effect. The major structural components contributed to strengthening include the high density of grain boundaries, dislocations and dislocation structures, and deformation twins. In the SS-USSR sample, the grain size for the GS surface layer is gradually increased from 153.5 nm in the topmost surface layer to 12.5 μm at a depth of over 1.1 mm, which is much smaller than that of the as-received sample. The decreased grain size gives rise to more grain boundaries. It has been proven that the grain boundaries are a strong obstruction to dislocation motion [33]. Hence, decreasing grain size can greatly strengthen the metals, giving rise to well-known refinement strengthening. This strengthening effect can be well described by the Hall-Petch relationship. Bahmanpour et al. [39] found that the strength of Cu-Zn alloy increased with decreasing grain size following the Hall-Petch relationship until the grain size was less than a critical value of 35 nm. The grain size of the present GS Cu-38Zn alloy is much larger than this critical value, thus softening behavior induced by the decreased grain size is not encountered. This suggests that the yield strength continuously decreases layer-by-layer from the outermost layer for the SS-USSR sample. Commonly, the yield strength is positively associated with hardness [23]. The continuously decreased hardness with the increasing depth for the SS-USSR sample (Figure 5) provides sound evidence for the enhancement in strength.

The observed twins also contribute to high strength. Our TEM observation reveals that the twin lamellae prevail in the gradient layer at a depth range of 100–200 μm, as indicated in Figure 4. The twin boundaries can strengthen metals by blocking dislocation motion [40,41,42]. Lu et al. reported the strength of the nanotwinned Cu increased with decreasing twin thickness until 15 nm following the Hall-Petch relationship [42,43]. Here, the observed twin lamellae have a small thickness (38.2 nm and 57.3 nm at the depth of 100 μm and 200 μm), thus greatly strengthening the GS surface layer. The activation of twins is related to the low stacking fault energy of the Cu-38Zn alloy [44,45]. The high density of dislocation can strengthen the metals through the work-hardening effect. According to Taylor’s model, the dislocation strengthening effect is proportional to the square root of total dislocation density [46,47,48]. Here, the high density of dislocations and dislocation structures fill the whole GS surface layer (Figure 2 and Figure 3) and thus induces a significant strength enhancement.

It has been proven that the incompatible deformation induces a synergistic strengthening effect in the GS metals, namely, hetero-deformation-induced (HDI) strengthening [37,38]. The incompatible deformation is resulted by the different mechanical properties of different layers in GS surface layer and is accumulated by geometrically necessary dislocations (GNDs). The synergistic strengthening or HDI strengthening renders the strength to exceed the predicted value using the rule of the mixture and is regarded as a significant strength contributor. Li et al. reported that the thick GS layer produced a higher synergetic strengthening effect [23]. Here, the GS surface layer is as thick as 1.1 mm and thus is helpful to develop the HDI strengthening for high strength. In short, the structural components and synergistic strengthening effect significantly contribute to the enhanced strength of the GS surface layer. The high yield strength of the free-standing 350-μm-thick gradient layer proves the high strength of the GS surface layer to a certain extent. This view leads us to believe that the strength of the USSR alloy increases with the increasing volume fraction of the GS layer. Thus, the DS-USSR sample with a high-volume fraction of GS surface layer (100%) is stronger than the SS-USSR sample.

The good uniform ductility of the USSR sample mainly stems from the high strain hardening rate, which suppresses plastic instability. For the SS-USSR sample, the volume fraction of the ductile coarse-grain layer is still 31.2%, which allows further dislocation accumulation during the tensile deformation and thus provides good ductility for the whole sample. For the DS-USSR sample, the volume fraction of the gradient layer reaches 100%, leading to low ductility. In the GS metals, HDI hardening is a significant contributor to the improved strain hardening capability and ductility through accumulating GNDs [49,50]. Moreover, the different layers within the GS surface layer may undergo different deformation mechanisms due to their different mechanical properties, and induce an extra strain hardening mechanism [51,52].

## 5. Conclusions

In the present work, a 1.1-mm-thick GS surface layer without surface damage was successfully produced in the Cu-38Zn alloy by using USSR at room temperature. This GS surface layer is much thicker than the previously reported one in the literature. The grain size for the GS surface layer gradually decreases from 153.5 nm in the topmost surface layer to 12.5 μm at a depth of over 1.1 mm. After single-sided USSR processing, yield strength increased from 297.4 MPa to 464.7 MPa, while a good uniform elongation of 10.7% is held which is far beyond that of the homogenous nano-grained and ultrafine-grained Cu-Zn alloys reported in literature, giving rise to a superior strength-ductility balance. After double-sided USSR processing, the strength is further improved but the ductility is decreased due to the increased volume fraction of the GS surface layer. The high strength of the GS surface layer mainly stems from the high density of grain boundaries, dislocations and dislocation structures, deformation twins, and GS-induced synergistic strengthening effect. The good ductility is majorly ascribed to the high strain hardening rate. This study also demonstrates that tailoring the volume fraction of the GS surface layer can tune the combination of strength and ductility in a certain range, which offers an approach to further optimize the strength-ductility combination of Cu alloy by regulating the USSR processing parameters in the future. Moreover, a thorough understanding of the deformation mechanism of the Cu alloy with a thick GS surface layer is worth expecting synthetically using advanced experimental methods, multiscale simulation, and theoretical studies.

## Figures and Tables

**Table 1:** Mechanical properties.

	Yield Strength (MPa)	Ultimate Tensile Strength (MPa)	Elongation (%)	Uniform Elongation (%)
As-received	297.4 ± 30.4	444.0 ± 15.2	39.1 ± 5.2	28.3 ± 4.7
SS-USSR	464.7 ± 7.2	530.3 ± 0.6	22.5 ± 2.8	10.7 ± 1.4
DS-USSR	476.2 ± 0.9	553.6 ± 13.3	16.9 ± 1.1	5.2 ± 0.5

## Data Availability

The data presented in this study are available on request from the corresponding author.
